# Antibiotic resistance profiles and genetic characterization of *Salmonella enterica* from water supplies in Kaduna State, Northwest Nigeria

**DOI:** 10.1186/s12866-025-04527-x

**Published:** 2025-12-11

**Authors:** Olajoke Mofoluke Alalade, Joseph Baba Ameh, Isa Obansa Abdullahi, Clement M. Z. Whong, Habiba Iliyasu Atta

**Affiliations:** 1 Department of Food Science and Technology, Aliko Dangote University of Science and Technology, Wudil, Kano state Nigeria; 2 Department of Microbiology, Ahmadu Bello University, Zaria, Kaduna state Nigeria

**Keywords:** Drinking water, Salmonella enterica, 16S rRNA sequencing, Kaduna, Nigeria, Antibiotic resistance genes.

## Abstract

**Background:**

Communities across Kaduna State, Nigeria, depend on diverse water sources, and the presence of *Salmonella enterica* is particularly concerning when the bacteria are resistant to antibiotics and possess resistance genes. The One Health approach recognizes that water quality, antimicrobial resistance patterns, and human health are closely linked, yet significant knowledge gaps exist regarding both the resistance patterns and the underlying genetic mechanisms of *Salmonella* in local drinking water sources of Kaduna state. This study aimed to determine the phenotypic antibiotic susceptibility patterns and detect some resistance genes in *Salmonella enterica* isolated from various drinking water sources in Kaduna State.

**Methodology:**

Five hundred sources of water used for drinking in six selected Local Government Areas of Kaduna state were sampled from 2014 to 2015. The samples were processed using standard bacteriological methods to isolate and identify *Salmonella* species, followed by molecular confirmation through 16 S rRNA gene sequencing. The consensus sequences of the isolates were subjected to BLAST in the GenBank of the National Center for Biotechnology Information (NCBI). The isolates were subjected to antibiotic susceptibility tests and investigation of some resistance genes were assessed.

**Results:**

Six isolates (1.2% isolation rate) were obtained from various sources and were identified as *Salmonella enterica*. The sequences were submitted to the NCBI GenBank and have been assigned accession numbers. Four (66.7%) of the isolates were resistant to tetracycline, nalidixic acid and sulfamethoxazole-trimethoprim, while 2 (33.3%) were pan-susceptible. One isolate was resistant to three (3) different classes of antibiotics. Antibiotic resistance genes –*tetA* and *sul1* were both detected in two isolates, obtained from treated pipe borne and well water respectively. The genes detected correlate with the phenotypic resistance observed.

**Conclusion:**

Antibiotic-resistant *Salmonella enterica* in drinking water poses a critical One Health threat, linking human, animal, and environmental health risks. The correlation between resistance genes and phenotypic patterns indicates antibiotic misuse in the study area at the time, creating reservoirs for multidrug-resistant pathogens and horizontal gene transfer. Urgent implementation of multi-sectoral One Health surveillance, strict antibiotic regulation, improved water treatment, antimicrobial stewardship programs, and rapid response protocols is essential across Kaduna state and Nigeria.

## Background

In developing countries such as Nigeria, waterborne diseases contribute greatly to the high rates of morbidity and mortality. These regions are plagued with issues such as an inadequate potable water supply, poor sanitation, open defecation, lack of personal hygiene and antimicrobial resistance. These factors play a major role in the incidence of outbreaks of diarrhoeal illnesses, which cause death particularly in children, and the immunocompromised individuals in the population [1, 2]. Diarrhoeal conditions, with approximately 90% linked to inadequate water quality, hygiene practices, and sanitation systems (WASH), represent a major contributor to illness and death among children under five in developing nations. This health challenge claims more young lives than HIV, malaria, and measles collectively. WASH programs are designed by the World Health Organization, to interrupt and manage the pathways through which bacterial transmission occurs [3].

Climate change, population explosion, increased demand, and mismanagement of water sources in recent decades have underscored the global shortage of clean water and has caused widespread water shortages in many regions, including Nigeria [4]. Across many states in Nigeria, the public water infrastructure has achieved limited success in satisfying residential and commercial water demands. Numerous households, especially those with lower incomes, resort to buying water from private suppliers at significantly inflated costs compared to public rates. Where water services do operate, they usually demonstrate unreliability, substandard quality, and lack long-term viability due to challenges in administrative oversight, operational procedures, tariff structures, and inadequate cost recovery mechanisms. Furthermore, many public water distribution networks have been shown to be degraded, with existing infrastructure capacity remaining underutilized as a consequence of insufficient maintenance practices and operational funding shortfalls [5].

In the urban and rural areas of Kaduna state, Nigeria, the problem of inadequate water supply persists. This has resulted in residents seeking alternative sources of water such as hand-dug wells, boreholes, surface water, and commercially available packaged water [6]. Sanitation is also quite poor in this location, as studies showed that 30% of the population practices open defecation and that the practice of improper sewage disposal is widespread [7, 8]. This is also similar to the findings of a research carried out in the neighboring Plateau state, where up to 44% of the respondents stated that the supply from the municipal pipe borne water was inadequate [9]. These problems persist in neighboring states such as Katsina, Kano and Niger [10–12].

*Salmonella* species are a genus of Gram negative rods, belonging to the family, *Enterobacteriaceae*. The genus comprises only two species, *Salmonella bongori* and *Salmonella enterica* with over 2,500 serovars identified to date, including Dublin, Typhi, Typhimurium, and Enteritidis [13, 14]. Salmonellae are ubiquitous and resilient bacteria that can survive several weeks in dry environments, and several months in water [13]. They have also been demonstrated to thrive in diverse environments, including environments with elevated temperatures and high organic matter contents [15]. *Salmonella* species can contaminate water and food sources in many ways, including through the runoff of agricultural and industrial wastes into water bodies, open defecation, and inadequate water treatment [16]. The detection of *Salmonella enterica* in drinking water is highly important because the organism has been implicated in outbreaks of typhoid fever, salmonellosis and gastroenteritis in humans and animals [13].

*Salmonella enterica* serovar Typhi is the causative agent of typhoid fever with symptoms ranging from fever and gastroenteritis, to organ failure, shock and death. It is a human-specific serotype [15]. The Typhimurium serovar, a non-typhoidal strain, gives rise to highly invasive cases of bacteremia leading to death especially in people living with HIV/AIDS [17].

These organisms have also been shown to harbor antibiotic resistance genes, becoming repositories of antibiotic resistance in the environment. The identification of these genes demonstrates the capacity for resistance dissemination through horizontal gene transfer, potentially expanding the antimicrobial resistance burden within microbial communities [18, 19]. The problem of antibiotic resistance continues to be a leading cause of concern to scientists and policy makers for a myriad of reasons, which include higher morbidity and mortality rates among infected individuals, and the resulting financial implications [20–22]. The World Health Organization has classified *Salmonella* as an organism of high priority with respect to antibiotic resistance [23].

Antimicrobial resistance (AMR) has emerged as one of the most significant global health threats of the 21 st century, with recent comprehensive analyses projecting 1.91 million deaths caused directly to bacterial AMR by 2050, representing a 67.5% increase from the levels reported in 2021 [24]. Within this global crisis, aquatic environments and wastewater have been increasingly recognized as significant routes for the spread and selection of antibiotic resistance genes and antibiotic-resistant bacteria [25].

The detection of antibiotic resistance genes in drinking water demonstrates how environmental, animal, and human health challenges are fundamentally connected, this is an important issue that the One Health approach seeks to address [26]. This connection between water contamination and antimicrobial resistance in Kaduna State directly contributes to the global antimicrobial resistance burden through the environmental dissemination of resistant pathogens and resistance genes. Furthermore, indiscriminate use of antibiotics, self-prescription, and the misuse of antibiotics in agriculture and animal husbandry has led to the contamination of the environment by residues of antibiotics, thereby worsening the problem of antibiotic resistance. There have been reports of increase in the frequency of isolation of drug-resistant *Salmonella* from water sources in Nigeria [27], therefore continuous monitoring of the drinking water sources for pathogens and antibiotic resistance is important. This study therefore is aimed to detect *Salmonella* species from drinking water sources in selected Local Government Areas of Kaduna State, Northwestern Nigeria, determine their susceptibility to commonly used antibiotics and finally screen the isolates for the presence of some antibiotic resistance genes.

## Methods

### Study area

**Fig. 1:**
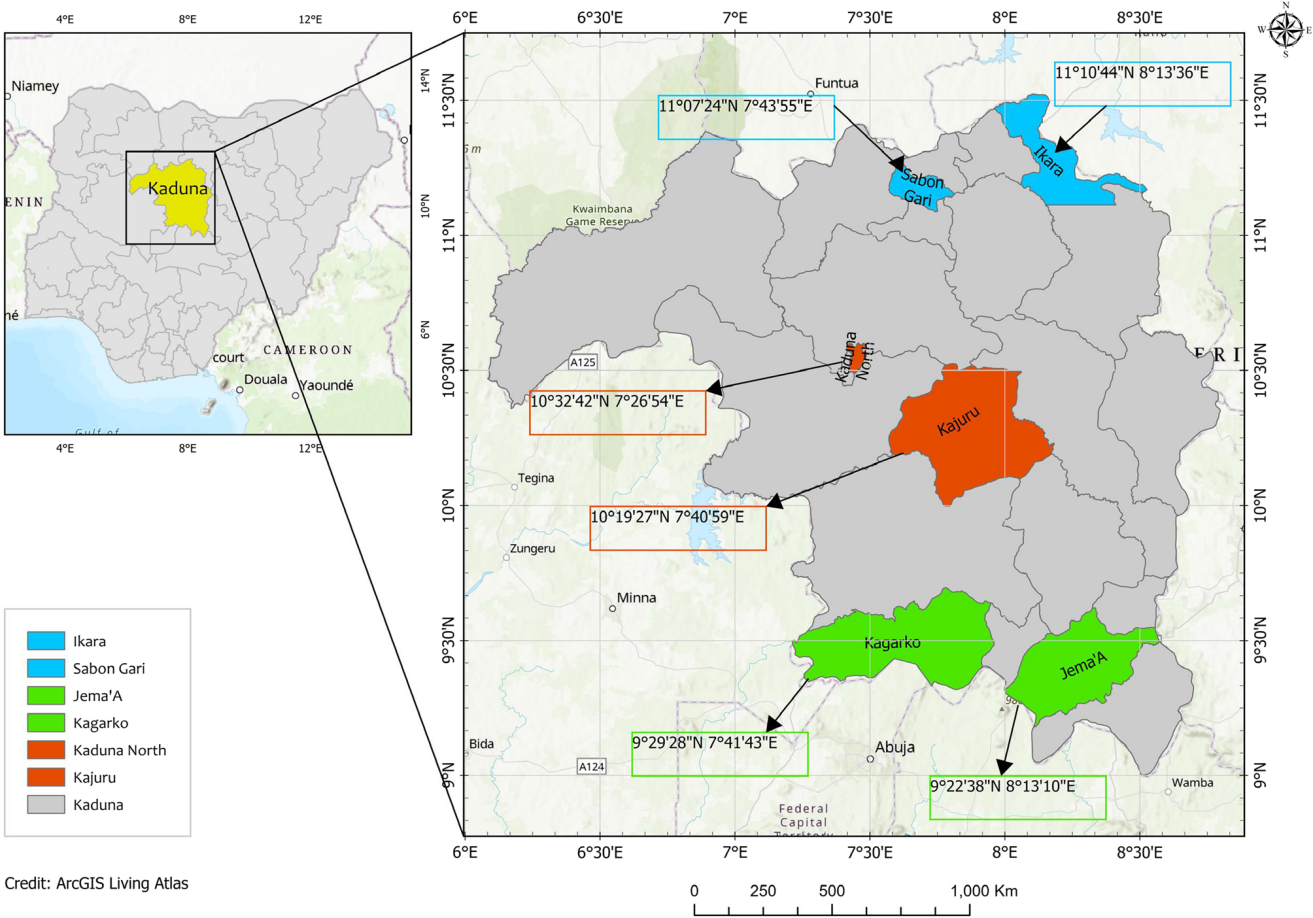
Map of Kaduna State showing study areas. The six LGAs selected for this study are highlighted in colors

Kaduna State was the study area, which lies in the center of Northwestern Nigeria and is approximately 960 km from the Atlantic Ocean. It is located at 10° 22’ 35.042’’ N and 7° 42’ 34.033 E [28]. The state is made up of twenty-three (23) Local Government Areas (LGA), grouped into three clusters namely the Kaduna North, Kaduna Central and Kaduna South senatorial districts. A simple random sampling technique without replacement was used to select two (2) LGAs in each of the three senatorial districts, ensuring that no adjacent LGAs were simultaneously included. The LGAs selected were: Jema’a and Kagarko LGAs for Kaduna South, Kajuru and Kaduna North LGAs for the Kaduna Central senatorial district and Ikara and Sabon-gari LGAs for Kaduna North Senatorial district (Fig. 1).

### Sample collection

Water samples were collected from the headquarters of each LGA (sample distribution is shown in Table 1) via a convenience sampling technique. Samples of drinking water were collected on the basis of availability at the time of sampling (March 2014–February 2015). We acknowledge that the temporal gap between sample collection and reporting represents a significant limitation regarding the current AMR situation. These findings should be interpreted as baseline historical data from the study period.

Five hundred samples of drinking water sources namely: commercially packaged (sachet), municipal treated pipe-borne water, wells, boreholes and streams were collected. One (1 L) of each sample was collected using sterile, wide-mouthed 1 L bottles, following standard methods as previously described [29–31] and immediately transported under cold storage to the postgraduate laboratory of the Department of Microbiology, Ahmadu Bello University, Zaria within 6 h of collection.

**Table 1:** Distribution of water samples collected from various local government areas (LGAs)

LGA	Borehole	Streams		Taps	Wells	Packaged water	Total
Sabon-gari	20	3		11	26	28	88
Ikara	24	3		5	29	19	80
Kaduna North	19		3	20	22	19	83
Kajuru	31	0		0	48	3	82
Jema’a	16	3		24	38	6	87
Kagarko	20	3		0	48	9	80
	**130**	**15**		**60**	**211**	**84**	**500**

### Isolation and Identification of *Salmonella enterica*

Each water sample was analyzed individually by inoculating 10 ml of each sample into 10 ml of double strength selenite F broth (TM Media, India) and incubated at 37 °C for 18 h (32, 33). After this period, a loopful of the enrichment culture showing growth (turbidity and reddish colouration) was selectively streaked on *Salmonella-Shigella* agar (SSA) (TM Media, India), and incubated at 37 °C for 18 h [30, 32, 33]. Suspected *Salmonella* colonies appeared colourless with or without dark centres. The isolates were purified by restreaking to obtain pure cultures which were stored on nutrient agar slants at 4 °C until use. The isolates were subjected to Gram-staining and biochemical tests (urease, triple sugar iron, motility, citrate, indole, methyl red, Voges-Proskaeur tests) via the Microgen^®^ GN-ID A *Enterobacteriaceae* identification kit (Oxoid, UK). The isolates that were presumptively identified to be *Salmonella* were tested further with the *Salmonella* latex agglutination kit (Microgen, England) by following the manufacturer’s instructions. The isolates that showed agglutination reaction with the test reagent were serologically confirmed to be *Salmonella* species. The pure isolates were stored on nutrient agar slants pending further tests.

### Molecular Characterization of *Salmonella* Isolates.

#### Extraction of genomic DNA

A single colony of a pure culture of each isolate selected for the polymerase chain reaction (PCR) was inoculated into 10 ml of Luria-Bertani (LB) broth (Merck, Germany) and incubated at 37 °C for 18 h. The overnight culture was aseptically streaked on freshly prepared nutrient agar to obtain discrete colonies. DNA was extracted using a modified phenol-chloroform method as previously described [34]. Briefly, the colonies were transferred into Eppendorf tubes containing 400 µL of lysis buffer for cell lysis. Specifically, 40 µL of proteinase was added to 200 µL of each isolate, followed by vortexing. The tubes were incubated at 65 °C for 10 min. After this, 400 µl of phenol chloroform was added and the tubes were vortexed to mix. The samples were centrifuged at 13,000 rpm for 10 min, and the upper layer was transferred into freshly labeled tubes. Each tube then received 400 µL of chloroform, was vortexed, and centrifuged again.

To the upper layer, 1 ml of absolute ethanol and 40 µl of 3 M sodium acetate were added and the tubes were stored at −20 °C overnight. After the incubation period, the tubes were centrifuged at −4 °C for 1 h after which the ethanol was discarded. Then, 400 µl of 70% ethanol was added and the tubes were centrifuged at 13,000 rpm for 10 min. The ethanol was then discarded and the DNA was subsequently air-dried. Then, 50 µl of DNase-free water was added and the DNA was stored at −20 °C until further use [34].

#### 16 S rRNA gene polymerase chain reaction and sequencing.

Polymerase chain reaction was carried out using the universal 16SrRNA primer (IDT, USA). Extracted genomic DNA (3 µL per Eppendorf PCR tube) were tested for purity and then combined with 5 µL of Master Mix (Promega, USA), 0.5 µL each of forward and reverse primers, and 1 µL of nuclease-free water in separate microtubes.

PCR amplification included the initial denaturation at 94 °C for 5 min. This was followed by 36 cycles of denaturation at 94 °C for 20 s; annealing at 56 °C for 30 s and extension at 72 °C for 45 s. The final extension was at 72 °C for 5 m and then a hold temperature at 4 °C [35]. Three (3) µl of the PCR product was electrophoresed in 2% agarose gel (Bioline) containing 5 µl of 10 mg/ml ethidium bromide at 100 V for 45 min. A 1 kb plus DNA marker (Invitrogen) was used as molecular size marker. DNA amplicons were examined under an ultraviolet transilluminator and the amplicons were viewed at around 1500 bp.

Sequencing was carried out with a sequencing machine (Applied Biosystems, HITACHI 3130 × 1 Genetic Analyzer). The sequences were analyzed with the Finch TV and BIOEDIT (version 7.2.5.0) software after which the Basic local alignment search tool (BLAST) was carried out on the National Centre for Biotechnology Information (NCBI) website (www.ncbi.nlm.nih.gov). The identity with the highest similarity was assigned to each isolate [36]. The sequences of the isolates from this study have been deposited in the Gen Bank and can be accessed at www.ncbi.nlm.nih.gov using the accession numbers assigned.

#### Antibiotic susceptibility testing of the isolates

Susceptibility testing was performed using Kirby Bauer disc diffusion assay on Mueller-Hinton agar (Lifesave Biotech, UK) as described by Clinical Laboratory Standards Institute (CLSI) [37]. The organisms were tested in vitro for susceptibility to the following commonly used antibiotics in the study area: amoxicillin (10 µg), ampicillin (10 µg), augmentin (30 µg), cefotaxime (30 µg), ciprofloxacin (5 µg), chloramphenicol (30 µg), gentamicin (10 µg), nalidixic acid (30 µg), tetracycline (30 µg) and sulfamethoxazole-trimethoprim (25 µg). The plates were incubated at 37 °C for 18–20 h. The diameter of each zone of inhibition was measured in millimeters [37]. The zone diameters were compared with the published limits of the CLSI. Each isolate was classified as sensitive (S), intermediate (I) or resistant (R). Organisms that were observed to be resistant to at least three different classes of antibiotics were classified as being multidrug resistant [38]. The Multiple Antibiotic Resistance (MAR) index was calculated by dividing the number of antibiotics an organism was resistant to by the total number of antibiotics tested [39].

#### Amplification of *tetA, tetB, sul1* and *blaTEM* genes via polymerase chain reaction.

Specific primer sets (Table 2) were used in separate PCR reactions to detect the tetracycline resistance genes (*tet*A and *tet*B), sulphonamide resistance gene (*sul*1) and β-lactamase gene (*bla*_TEM_). The primers were synthesized by Inqaba biotech, South Africa. They were all diluted following the manufacturer’s instructions to produce working solutions for the PCR. Each 10 µL PCR reaction mixture contained 5 µL of Master Mix (Promega, USA), 1 µL of nuclease-free water, 3 µL of template DNA, and 0.5 µL of each primer mix. A tube containing all the above, but excluding the DNA template was also included to serve as the negative control.

PCR amplification included the initial denaturation at 94 °C for 5 min, followed by 36 cycles of denaturation at 94 °C for 20 s; annealing at 56.0, 56.5, 64.5 and 60.4 °C for *tet*A, *tet*B, *sul*1 and *bla*TEM respectively for 30 s and extension at 72 °C for 45 s. The final extension was at 72 °C for 5 m and then a hold temperature at 4 °C. The PCR protocol was carried out as previously reported [35, 36, 40–42]. Three (3) µl of the PCR product was electrophoresed in 2% agarose gel (Bioline) containing 5 µl of 10 mg/ml ethidium bromide at 100 V for 45 min. A 1 kb plus DNA marker (Invitrogen) was used as molecular size marker. DNA amplicons were examined under an ultraviolet transilluminator and the results were documented [35–37].

**Table 2:** Primers used for molecular characterization and antibiotic resistance genotyping

Primer	Primer sequence (5’−3’)	Size (bp)	Anealing temp (°C)	Reference
16SrRNA	F-AGA GTT TGA TCA TGG CTC AG R-AAG GAG GTC ATC CAA CCG CCA	1500	56.0	[36]
*tet*A	F-GGTTCACTCGAACGACGTCA R-CTGTCCGACAAGTTGCATGA	577	56.0	[35]
*tet*B	F-CTC AGT ATT CCA AGC CTT TG R-ACT CCC CTG AGC TTG AGG GC	415	56.5	[40]
*sul*1	F-TGA GAT CAG ACG TAT TGC GC R-TTG AAG GTT CGA CAG CAC GT	406	64.5	[40]
*bla*_TEM_	F-GCG GAA CCC CTA TTTG R-ACC AAT GCT TAA TCA GTG AG	964	60.4	[41]

Key: Tm- annealing temperature; 16 S rRNA- 16 S ribosomal RNA gene, *tet*A and *tet*B- Tetracycline resistance genes A and B; *sul*1- sulfonamide resistance gene 1, *bla*_TEM−_ beta lactamase temoniera gene

## Results

Six (6) isolates were obtained in this study, giving an isolation rate of 1.2%. The isolates were observed to appear as colorless colonies with black centers on *Salmonella- Shigella* agar. They were observed to be Gram negative rods after Gram staining. The biochemical and agglutination characteristics of the isolates obtained presumptively identified the organisms as *Salmonella enterica*. The isolates were oxidase negative, urease negative, they fermented glucose and produced Hydrogen sulphide gas among others. Of the six isolates obtained, 4 (66.7%) were obtained from well water samples. Two of these six (33.3%) were isolated from wells in Ikara, and one (1) each from wells in Sabongari and Jema’a LGAs (16.6% each). The other 2 isolates were isolated from a borehole and treated pipe-borne water sources in Jema’a LGA.

**Fig. 2:**
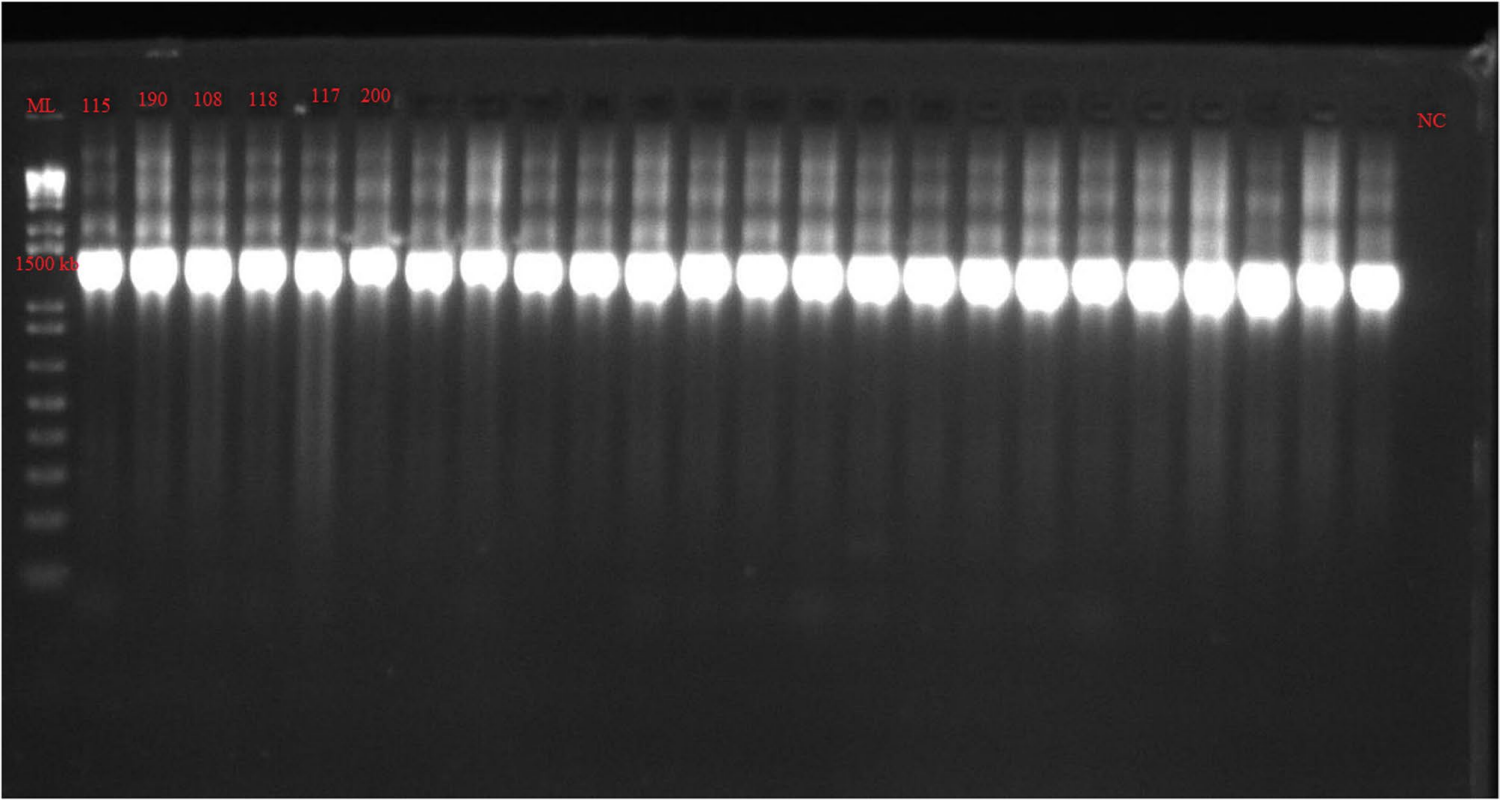
Gel image of 16 S rRNA gene amplicons in *Salmonella enterica* isolates. The gene was amplified at 1500 bp as indicated on the molecular ladder. All six isolates obtained in this study contained the 16 S rRNA gene. ML- 1kb plus molecular ladder, NC- Negative control, Lanes numbered: *Salmonella* isolates, all positive for the 16S rRNA gene

Figure 2 is the gel image of the 16 S rRNA gene of the isolates which was amplified at 1500 bp. The 16 S rRNA sequences were subjected to BLAST on the National Center for Biotechnology Information (NCBI) website. The accession numbers assigned to the sequences obtained in this study are listed in the Table 3.

**Table 3:** Resistance patterns and genes in *Salmonella enterica* isolates from water sources in Kaduna state

Isolate code	Source	Antibiotic resistance pattern	Antibiotic resistance gene detected	GenBank accession numbers
115JM	Borehole	TE	nil	KT737738
190IK	Well water	Susceptible to all antibiotics tested	nil	KU255189
108SG	Well water	TE	*TetA*	KT737737
118JM	Treated Pipe borne water	TE, NA, SXT	*Tet A*,* Sul1*	KT737736
117JM	Well water	TE	*Tet A*,* Sul1*	KU255190
200IK	Well water	Susceptible to all antibiotics tested	nil	KU255191

KEY IK-Ikara, JM-Jema’a, KG-Kagarko, SG-Sabon Gari, KJ-Kajuru, KN-Kaduna North LGAs, TE- Tetracycline, NA- Nalidixic acid, SXT- Sulfamethoxazole trimethoprim

Table 3 shows the antibiotic resistance patterns and genes detected in the six *Salmonella enterica* isolates obtained from this study. The isolates from Ikara LGA were susceptible to all the antibiotics tested, whereas the one from pipe-borne water from Jema’a (118JM) was resistant to three classes of antibiotics and is multidrug resistant. The highest resistance observed was to tetracycline alone (50%), while 1 isolate showed a TE, NA, SXT resistance pattern. The Multiple Antibiotic Resistance (MAR) index of the isolates ranged from 0.1 to 0.3 (Table 4).

**Table 4:** Antimicrobial resistance patterns and multiple antibiotic resistance index of resistant isolates

Antimicrobial resistance pattern	Source of Isolates (*n*)	Isolates showing resistance pattern *n* (%)	No. of resistant AMs/classes	MARI
TE	Borehole (1), Well water (2)	3 (50)	1	0.1
TE, NA, SXT	Treated Pipe borne water (1)	1 (17)	3	0.3

KEY TE- Tetracycline, NA- Nalidixic acid, SXT- Sulfamethoxazole trimethoprim, AM- Antimicrobial, MARI- Multiple Antibiotic Resistance Index (No. of resistant antibiotics/Total no. of antibiotics tested).

**Fig. 3:**
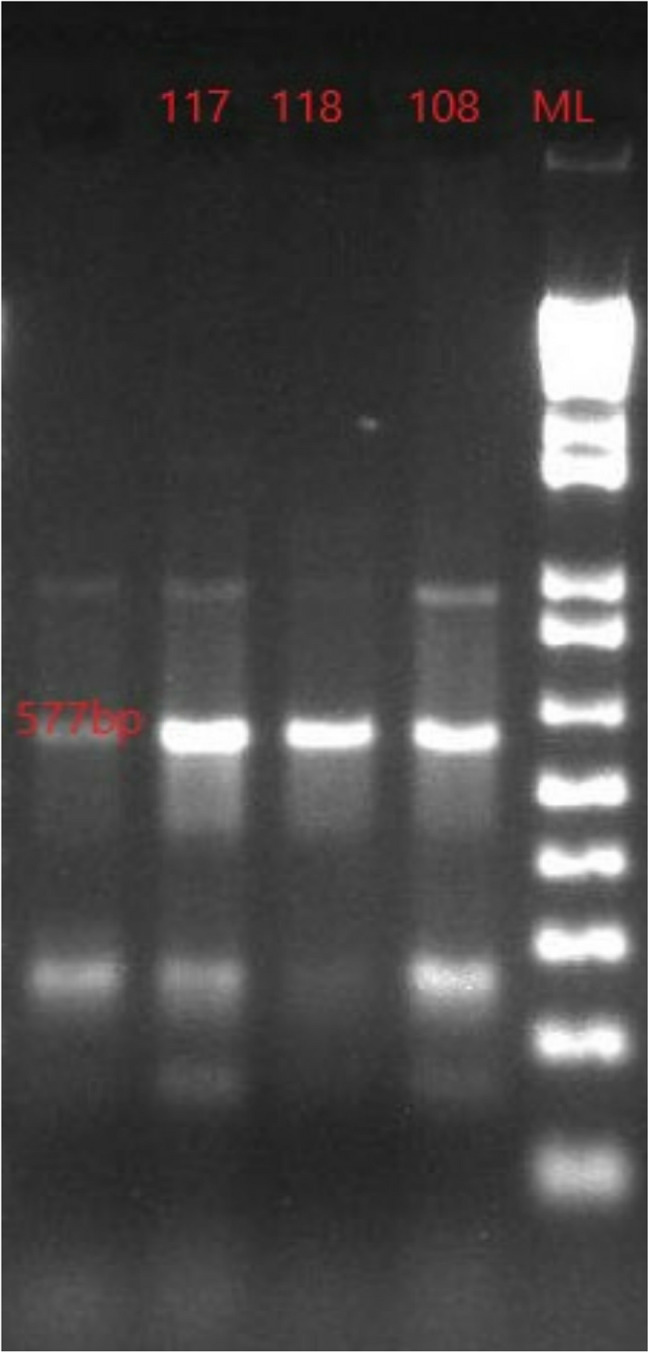
Gel image of *tet*A gene amplicons in *Salmonella enterica *isolates. The gene was amplified at 577 bp as indicated on the molecular ladder. Three isolates contained this gene (108SG, 117JM and 118JM). ML- 1kb plus molecular ladder, NC- Negative control, Lanes numbered: *Salmonella*isolates positive for the *tet*A gene

**Fig. 4:**
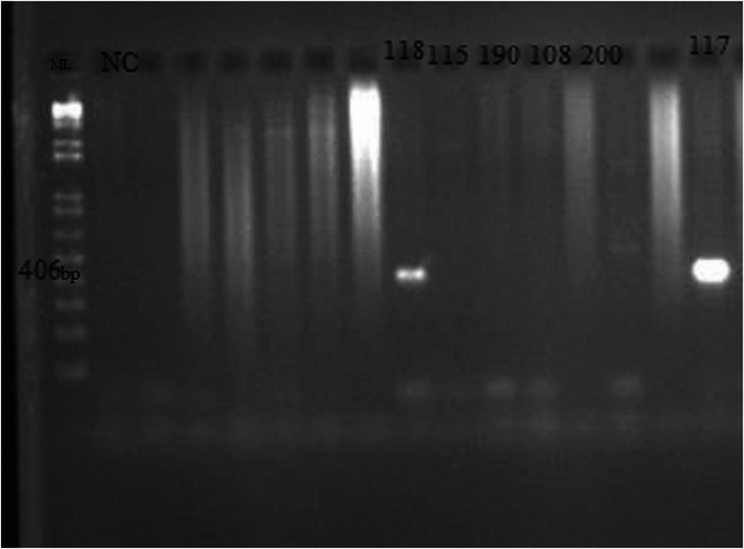
Gel image of *sul*1 gene amplicons in *Salmonella enterica *isolates. The gene was amplified at 406 bp as indicated on the molecular ladder. Two isolates contained this gene (117JM and 118JM). ML- 1kb molecular ladder, NC- Negative control, Lanes numbered: *Salmonella enterica *isolates positive for the *sul*1 gene

The *tet*B and *bla*TEM genes were not amplified from any of the isolates, whereas the *tet*A and *sul*1 genes were detected in 3 and 2 isolates respectively. The gel images of the amplified *tet*A and *sul*1 genes are shown in Figs. 3 and 4. The amplicons were observed at 577 and 406 bp respectively.

## Discussion

This study aimed to evaluate the prevalence of *Salmonella* species, characterize their phenotypic antibiotic susceptibility patterns, and detect selected resistance genes in *Salmonella enterica* strains isolated from various drinking water sources in Kaduna State, Northern Nigeria. The isolation and characterization of *Salmonella enterica* in drinking water is of public health concern. Four out of the six isolates obtained were isolated from well water samples. This suggests that wells in the study area may serve as important reservoirs for *Salmonella* likely due to reasons such as proximity to pit latrines and septic systems, inadequate well construction and maintenance, surface water infiltration during rainfall and poor sanitation practices in surrounding areas [42]. A number of other studies have reported the incidence of these organisms in similar sources of water in Kaduna state [43, 44], Benin city [45], other parts of Africa and beyond [46–49]. This similarity across Nigerian states and other parts of Africa suggests that there are similar antibiotic use practices, sanitation challenges, and environmental contamination pathways across the region.

The isolation of *Salmonella enterica* from water sources similarly indicates fecal contamination and suggests that the water is not safe for drinking. Ingestion of these bacteria in drinking water can lead to symptoms such as fever, stomach cramps, vomiting, stooling and other more serious consequences of typhoid [46, 48]. Drinking water has also been identified as a risk factor for typhoidal illness in the study area [49]. A study carried out in Kaduna State showed that residents who use well water had a higher prevalence of typhoid fever compared to those who use other sources of water [50]. This corroborates with our findings which show that 66.7% of our isolates were obtained from well water.

Notably, *Salmonella enterica* was isolated from treated pipe-borne water (Table 3), which is supposed to have undergone filtration and chlorination in water treatment plants before being distributed to homes and communities. In these cases, chlorination may be inadequate or the pipes in the distribution line may have become old and incompetent, thereby becoming a point of contamination of the treated water [51]. The isolate obtained from treated pipe-borne water was resistant to 3 antibiotics of different classes (MARI = 0.3) and can be said to be a multidrug resistant strain (Table 4) [19]. Some of the organisms reported in this study have been previously reported to contain the *inv*A gene [30] which is a virulence gene and an indication of their likely pathogenicity. Our findings are an indication that the water treatment in the study area may be inadequate hence the isolation of *Salmonella* in pipe-borne water.

The detection of the *tet*A and *sul*1 genes in our isolates (Table 3; Figs. 2 and 3) agrees with a study done in North-central Nigeria, where the isolates obtained from drinking water, poultry and their drinking water harbored these and other genes [27]. These genes encode for resistance to tetracycline and sulfamethoxazole-trimethoprim respectively. Tetracyclines are broad-spectrum antimicrobials used to treat and prevent diseases caused by both Gram positive and negative bacteria in humans, animals and even in plants [52]. The *tet*A gene is one that codes for efflux pumps which reduce the accumulation of tetracycline inside a bacterial cell by expelling the antibiotic out of the cell at the expense of a proton [53]. The *tet*A gene facilitates bacterial survival in presence of tetracycline via efflux, often plasmid-borne, enabling horizontal transfer [54]. The *sul*1 gene encodes an altered version of dihydropteroate synthase (DHPS) that has a low affinity for sulfonamides which target the folate synthesis pathway [55]. The *sul*1 gene allows bacteria to continue folate synthesis even in presence of sulfonamides, promoting survival and is often associated with integrons and co‐resistance.

Interestingly, both genes were detected in the isolates (117JM and 118JM) obtained from treated pipeborne water and well water sampled from Jema’a LGA. This could be an indication of the persistence of Salmonella even after treatment, or that the treatment in the study area was ineffective. Furthermore, these findings show that there could be issues of gross misuse of antibiotics in Jema’a LGA. On the other hand, the 2 isolates obtained from Ikara LGA were pan-susceptible. The geographic variation observed in resistance patterns of the isolates, with pan-susceptible isolates from Ikara LGA versus resistant isolates from Jema’a LGA, may reflect differences in antibiotic usage patterns, sanitation infrastructure, and environmental contamination levels across regions.

Out of the 6 isolates obtained, 4 (66.6%) were resistant to tetracycline. The rise in reports of antibiotic resistant bacteria in developing countries such as Nigeria have been linked to various factors such as misuse of antibiotics, self-prescription and over the counter sale of antibiotics without any prescription, and indiscriminate use of antibiotics as prophylactic treatment of farm animals [50]. The high resistance rate to tetracycline observed in this study is similar to the results of a study conducted on *Salmonella* strains obtained from food and animals [54]. The most common tetracycline resistant gene observed in their study was *tet*A [54] which agrees with our findings. Sulfamethoxazole-trimethoprim is a sulfonamide which shows broad- spectrum activity against bacteria that do not possess mechanisms to overcome its inhibition effects [55, 56]. The *sul*1 gene is one of the most commonly observed genes responsible for sulfonamide resistance, as stated in a review of articles written between 2009 and 2019 on tetracycline and sulfonamide resistance in *Salmonella* spp [57]. These antibiotics are first-line antibiotics that are usually available over the counter, leading to misuse in the region [38].

In our study, there was a high correlation between the phenotypic and genotypic antibiotic resistance to tetracycline, as it was observed that 3 out of the 4 isolates resistant to the antibiotics, carried the *tetA* gene. One of the isolates (115JM) was resistant to tetracycline, but did not harbor the *tet*A gene. This could be due to the presence of other *tet* genes which were not detected in this study. Also, another isolate (117JM) harbored the *sul*1 gene but did not show phenotypic resistance to Sulfamethoxazole-trimethoprim. This could be because the presence of a gene may not necessarily be associated with corresponding phenotypic expression, as it could be a silent gene [27, 58]. It is worthy of note that *tet*B and *bla*TEM genes which are responsible for tetracycline and ampicillin resistance respectively, were not amplified in our isolates. The presence of *tetA* and *sul1* genes in 2014–2015 establishes that these mobile genetic elements were already circulating in environmental reservoirs a decade ago, which is relevant for understanding the evolutionary history of AMR in the region.

Other studies have shown that antibiotic misuse is rife in the study area [51, 59]. Multiple antibiotics resistance (MAR) index is a tool that reveals the spread of bacteria resistance in a given population. A MAR index greater than 0.2, as observed in one isolate, implies that the strains of such bacteria originate from an environment where several antibiotics are used. It also shows that the environment where such bacteria were isolated were high risk contamination sites [39].

A systematic review of studies on Salmonella in Nigeria from 1999 to 2018 revealed that over 60% of reported isolates developed resistance to two or more antibiotics, with ampicillin, sulfamethoxazole-trimethoprim, tetracycline, and amoxicillin being the most commonly resisted antibiotics [60]. According to Nigeria’s national antimicrobial resistance surveillance system data from 2019 to 2021, Salmonella demonstrated to 70–90% resistance to fluoroquinolones and 20–30% resistance to cephalosporins. This is an indication that even newer antibiotics are becoming compromised [60, 61]. The implication of this finding is that healthcare providers must rely on more expensive second-line or third-line antibiotics leading to increased healthcare costs and treatment duration for affected populations. These observed resistance patterns in water from various LGAs of Kaduna State mirror broader trends across Nigeria and Sub-Saharan Africa, indicating that local antibiotic use practices, combined with inadequate water and sanitation infrastructure, have created environmental reservoirs of resistant bacteria that pose significant public health risks.

The One Health framework has emerged as a strategic response to antimicrobial resistance challenges, recognizing that similar antimicrobial agents are used in fields such as human healthcare, veterinary medicine, and agricultural practices, while acknowledging that pathogenic organisms can colonize both human and animal hosts. This integrated approach emphasizes the connection between human, animal, and environmental health systems, highlighting the critical importance of comprehensively examining how environmental factors contribute to disease dynamics and pathogen transmission pathways [62]. The contamination of water sources with enteric pathogens like *Salmonella enterica* creates a food safety risk when the water is used in production of fresh produce such as vegetables. The contamination could occur through the use of such contaminated water for irrigation, through the use of manure or sewage sludge for fertilizer production [63]. When contaminated water is consumed by livestock or in food animal production facilities, it can colonize the animals’ gastrointestinal tracts. This establishes reservoirs of resistant bacteria in the food chain, leading to contaminated meat, dairy, and egg products. The animals themselves may not show symptoms but become silent carriers and shedders of the bacteria [64, 65].

*Salmonella* infections can be prevented by public enlightenment on proper hygiene, ensuring that cross-contamination does not occur. Furthermore, since the organism is heat-sensitive, boiling of drinking water can be encouraged in the study area [66]. The limitations of this research include the inability to collect water samples from all the LGAs of Kaduna state due to security challenges in some areas, and lack of funding. In our resource-limited settings at the time the research was done, the access to well-characterized resistant reference strains was limited. While we used published primer sequences, verified amplicon sizes, included negative controls in all reactions, and observed correlation between phenotypic and genotypic resistance, the lack of positive controls means we cannot definitively rule out non-specific amplification, although the exact size match and phenotype-genotype correlation make this unlikely. Future studies should ideally include characterized positive control strains or perform sequencing of resistance gene amplicons for definitive confirmation.

## Conclusion

To the best of our knowledge, this study represents one of the first molecular investigations of antibiotic-resistant *Salmonella enterica* in drinking water sources across various Local Government Areas of Kaduna State, Northwestern Nigeria. The isolation of *Salmonella enterica* which are resistant to antibiotics is a public health concern for the consumers as much of the sampled water is typically consumed without further treatment, increasing the risk of exposure to waterborne pathogens. The integration of phenotypic susceptibility testing with molecular detection of resistance genes (*tetA* and *sul1*) provides novel insights into the genetic basis of antimicrobial resistance in drinking water in the study area and highlights the need for effective monitoring and prevention strategies to safeguard drinking water sources in the study area.

The strong correlation between detected resistance genes and phenotypic patterns (75% concordance for tetracycline resistance) validates the utility of molecular surveillance in tracking AMR dissemination through water sources. This underscores the importance of incorporating drinking water monitoring into One Health surveillance strategies, as such monitoring would yield valuable insights into the dissemination patterns and environmental persistence of antibiotic-resistant bacteria and resistance genes. The use of molecular methods for identification provides a more rapid and accurate method of identifying these organisms in drinking water. The geographic variation in resistance patterns, with pan-susceptible isolates from Ikara LGA versus resistant isolates from Jema’a LGA, suggests localized differences in antibiotic selection pressure and environmental contamination that warrant further investigation.

This study contributes significantly to the limited body of literature on waterborne AMR in Northern Nigeria by providing baseline molecular data on resistance gene prevalence in drinking water, which is essential for tracking temporal trends and evaluating intervention strategies. It also documents the environmental dimension of AMR in a region where most previous research has focused on clinical or food-animal sources. Furthermore, infrastructural defects have been highlighted by the isolation of resistant bacteria from treated water, which has important implications for water management policies across Nigeria. Lastly, it supports the implementation of One Health surveillance frameworks by demonstrating how environmental monitoring can identify AMR threats before they translate into clinical infections and contributes to Nigeria’s National Action Plan on AMR by providing evidence-based data to inform water quality standards and antibiotic stewardship programs.

It is recommended that water for drinking purposes in the LGAs of Kaduna state that were studied, should undergo treatment such as boiling, as this is an easily accessible method of treatment and is effective in destroying *Salmonella enterica.* In addition, more effort needs to be made by all stakeholders in the study area to curtail the spread of antibiotic resistance in the study area. This includes the implementation of advanced water purification methods, protection of water sources to reduce the risk of contamination, and regular monitoring of water sources, livestock and the environment for antibiotic-resistant bacteria.

These findings, although limited in scope, underscore the need for routine monitoring of water sources in the study area for antibiotic resistant pathogens. While these findings reflect the AMR landscape from 2014 to 2015 and may not represent the current situation, they establish important baseline data and demonstrate the historical presence of antibiotic-resistant *Salmonella enterica* and mobile resistance genes in drinking water sources. Urgent follow-up surveillance using similar molecular approaches is needed to assess whether resistance patterns have intensified, whether new resistance genes have emerged, and whether interventions implemented during the past decade have had any impact on reducing AMR in water sources. It is also recommended that further studies should serotype the *Salmonella enterica* isolates from drinking water in Kaduna state, to provide more insight to the serotypes circulating in drinking water.

## Data Availability

The deposited sequences from this study can be accessed at https://www.ncbi.nlm.nih.gov with accession numbers KT737736, KT737737, KT737738, KU255189, KU255190 and KU255191. Other data used and/or analysed during the current study are available from the corresponding author on reasonable request.

## Abbreviations

BLAST: Basic Local Alignment Search Tool
CFU: Colony Forming Units
DNA: Deoxyribonucleic Acid
LGA: Local Government Area
MARI: Multiple Antibiotic Resistance Index
NCBI: National Center for Biotechnology Information
PCR: Polymerase Chain Reaction
rRNA: Ribosomal Ribonucleic Acid
SSA: Salmonella-Shigella Agar
WASH: Water, Sanitation and Hygiene
WHO: World Health Organization
